# 1-(4-Phenoxybenzyl) 5-Aminouracil Derivatives and Their Analogues – Novel Inhibitors of Human Adenovirus Replication

**Published:** 2018

**Authors:** N. A. Nikitenko, E. S. Gureeva, A. A. Ozerov, A. I. Tukhvatulin, F. M. Izhaeva, V. S. Prassolov, P. G. Deryabin, M. S. Novikov, D. Y. Logunov

**Affiliations:** N.F. Gamaleya Federal National Research Center for Epidemiology and Microbiology, Gamaleya Str. 18, Moscow, 123098, Russia; Volgograd State Medical University, Pavshih Bortsov Sq. 1,Volgograd, 400131, Russia; Engelhardt Institute of Molecular Biology, Russian Academy of Sciences, Vavilova Str. 32, Moscow, 119991, Russia

**Keywords:** Human adenovirus, 5-aminouracil derivatives, adenovirus replication, inhibitors of adenovirus replication

## Abstract

Adenovirus infections are characterized by widespread distribution. The lack of causal therapy, which is effective in treating this group of diseases, explains the need for new therapeutic drugs. Notably, anti-adenoviral activity of [4-(phenoxy)benzyl]-5-(phenylamino)-6-azauracil, 1-[4-(phenoxy)benzyl]-5-(morpholino) uracil, 1-[4-(4-chlorophenoxy)benzyl]-5-(morpholino) uracil, and 1-[4-(4-fluorophenoxy)-benzyl]-5-(morpholino) uracil was observed.

## INTRODUCTION

Human adenoviruses (HAdV) are nonenveloped viruses, and their genome is linear nonsegmented double-stranded DNA [1]. Adenovirus infections, which affect persons of all ages, are widespread and highly contagious. Human adenoviruses most often affect the mucous membranes of the respiratory tract [2, 3], eye [4], gastrointestinal tract [5], and genitourinary system [6]. The most dangerous manifestations of adenovirus infections occur in immunocompromised patients (recipients of hematopoietic stem cell transplant, HIV-infected individuals, etc.) [7, 8], in whom they can lead to the development of acute infectious diseases resulting in fatal outcomes [9].

At the moment, there are no selective chemotherapeutic agents that are highly effective against adenoviral infections [10]. Typically, a broad spectrum of antiviral agents is used, such as interferon or interferon inducers and corticosteroid medications [11]. However, interferon inducers are not effective enough, since adenoviruses are insensitive to interferon. The derivatives of acyclonucleotides, such as cidofovir, also display low activity; e.g., the use of (S)-1-(3-hydroxy-2-phosphonylmethoxypropyl) cytosine [12] is limited by its high nephrotoxicity [13]. Therefore, the development of low-toxic chemotherapy drugs that are effective against adenoviral infections remains relevant.

The purpose of our work was to study the inhibitory properties of new 5-amino derivatives of uracil [14] against human adenoviruses. Based on an analysis of the relationship between the structure and biological activity of uracil derivatives studied earlier [15], new 5-aminouracil derivatives which can presumably inhibit DNA-containing viruses were constructed and synthesized. It was shown that these compounds are highly effective in suppressing the replication of human adenoviruses *in vitro*. In addition, we studied the relationships between anti-adenoviral effect and the presence of various substituents in the structure. For example, we identified the key role of the aromatic fragment in the potency of the anti-adenoviral activity. Therefore, a new type of inhibitors of human adenovirus replication has been identified. The data obtained can contribute to the development of novel antiviral therapy *in vivo*.

## EXPERIMENTAL

The ^1^H and ^13^C NMR spectra were recorded on a Bruker Avance 400 spectrometer (Bruker, Germany) (400 MHz for ^1^H and 100 MHz for ^13^C) in DMSO-D_6_, with tetramethylsilane as the internal standard. Thin layer chromatography was performed on TLS Silica gel 60 F_254_ (Merck, Germany) plates using ethyl acetate as the eluent. The plates were developed using a UV lamp VL-6.LC (Vilber, France). Melting points were measured in glass capillaries on a Mel-Temp 3.0 instrument (Laboratory Devices Inc., USA).

The starting 5-(phenylamino) uracils and -6-azauracils were synthesized according to [16], 5-(morpholino) uracil – according to [17], 4- (phenoxy)benzyl bromides were obtained by brominating the starting 4-(phenoxy)toluenes with molecular bromine by irradiation with light in boiling chloroform in accordance with [14]. Synthesis of the starting 1-[ω-(phenoxy)- alkyl]-5-bromuracils was carried out by condensing equimolar amounts of 2,4-bis(trimethylsilyloxy)- 5-bromopyrimidine and 1-bromo-ω-(phenoxy) alkane through heating to 160–170 °C for 1 hour according to [18].

**The general method for the synthesis of 1-[4-(phenoxy)benzyl]-5-amino-6- azauracil (compounds 1, 2) and -uracil derivatives (compounds 3 - 5).**

A suspension of 4.90 mmol of 5-amino-6-azauracil or 5-aminouracil and 0.1 g (1.87 mmol) of NH_4_Cl in 30 ml of hexamethyldisilazane (HMDS) was boiled for 12 hours until a clear solution formed. Excess HMDS was removed under reduced pressure, the residue was dissolved in 50 ml of anhydrous 1,2-dichloroethane, and 4.94 mmol of 4-(phenoxy)benzyl bromide was added to the solution, after which the mixture was boiled for 24 hours while protected from air moisture. The reaction mass was cooled to room temperature, treated with 10 ml of isopropyl alcohol, evaporated under reduced pressure, and the residue was purified by flash chromatography, eluting with chloroform-methanol (10:1). The fractions containing the product were combined and evaporated to dryness under reduced pressure. The solid residue was recrystallized from ethyl acetate-hexane (2:1).

*1-[4-(Phenoxy)benzyl]-5-(phenylamino)-6-azauracil (1). *Yield 67%, *T*_mp_ 264–266°C, Rf 0.76 (ethyl acetate). ^1^H-NMR (DMSO-D_6_), δ, ppm: 4.95 (2H, s, CH_2_); 6.89–7.03 (5H, m, H-2’, H-3’, H-4’, H-5’, H-6’); 7.10 (1H, t, *J *= 7.1 Hz, H-4”’); 7.22 (2H, t, *J *= 7.6 Hz, H-3”’, H-5”’); 7.33 (2H, t, *J *= 7.7 Hz, H-3”, H-5”); 7.38 (2H, d, *J *= 8.2 Hz, H-2”, H-6”); 7.61 (2H, d, *J *= 7.8 Hz, H-2”’, H-6”’); 8.33 (1H, s, N3H); 13C-NMR (DMSO-D_6_), δ, ppm: 52.7; 119.0; 119.1; 119.3; 122.4; 123.9; 128.9; 130.3; 130.4; 132.5; 139.8; 140.0; 148.0; 154.7; 156.8; 157.3.

*1-[4-(Phenoxy)benzyl]-5-[(3,5-dichlorophenyl)amino]-6-azauracil (2). *Yield 56%, *T*_mp_ 224.5–226°C, Rf 0.78 (ethyl acetate). ^1^H-NMR-sprectrum (DMSO_6_), δ, ppm: 4.95 (2H, s, CH_2_); 6.93–6.98 (4H, m, H-2”’, H-4”’, H-6”’, NH); 7.10 (1H, t, *J *= 6.9 Hz, H-4’); 7.22 (2H, t, *J *= 7.6 Hz, H-3’, H-5’); 7.33 (2H, d, *J *= 7.5 Hz, H-2’, H-6’); 7.39 (2H, d, *J *= 8.2 Hz, H-3”, H-5”); 7.61 (2H, d, *J *= 7.8 Hz, H-2”, H-6”); 8.33 (1H, s, N^3^H); 13C-NMR-spectrum (DMSO-D_6_), δ, ppm: 31.1; 36.1; 40.3; 51.9; 116.9; 118.9; 120.9; 123.8; 130.3; 130.5; 132.0; 134.2; 139.6; 142.1; 147.9; 154.3.

*1-[4-(Phenoxy)benzyl]-5-(morpholino)uracil (3). *Yield 68%, *T*_mp_ 182–184°C, Rf 0.41 (ethyl acetate). ^1^H-NMR (DMSO-D_6_), δ, ppm: 2.82 (4H, s, 2 × CH_2_); 3.64 (4H, t, *J *= 4.4 Hz, 2 × CH_2_); 4.82 (2H, s, CH_2_); 6.96 (2H, d, *J *= 8.3 Hz, H-2’, H-6’); 6.98 (2H, d, *J *= 8.0 Hz, H-3”, H-5”); 7.13 (1H, t, *J *= 8.0 Hz, H-4’); 7.18 (1H, s, H-6); 7.32–7.36 (4H, m, H-3’, H-5’, H-2”, H-6”); 11.37 (^1^H, s, N^3^H). ^13^C-NMR (DMSO-D_6_), δ, ppm: 40.3; 50.2; 50.4; 66.3; 118.9; 119.0; 123.9; 127.2; 129.7; 130.2; 130.4; 132.3; 150.1; 156.5; 156.8; 161.2.

*1-[4-(4-Chlorophenoxy)benzyl]-5-(morpholino)uracil (4). *Yield 81%, *T*_mp_ 204–206°C, Rf 0.32 (ethyl acetate). ^1^H-NMR-spectrum (DMSO-D_6_), δ, ppm: 2.82 (4H, s, 2 × CH_2_); 3.64 (4H, s, 2 × CH_2_); 4.80 (2H, s, CH_2_); 6.80 (2H, d, *J *= 7.6 Hz, H-2’, H-6’); 7.12 (2H, d, *J *= 7.4 Hz, H-3’, H-5’); 7.39 (2H, d, *J *= 8.2 Hz, H-3”, H-5”); 7.61 (2H, d, *J *= 7.8 Hz, H-2”, H-6”); 7.70 (1H, s, H-6); 11.42 (1H, s, N^3^H). ^13^C-NMR-spectrum (DMSO-D_6_), δ, ppm: 50.1; 50.5; 67.0; 118.8; 121.1; 122.5; 123.2; 124.5; 134.0; 134.1; 138.5; 149.8; 154.2; 160.1; 164.2.

*1-[4-(4-Fluorophenoxy)benzyl]-5-(morpholino)uracil (5). *Yield 74%, *T*mp 220–222°C, Rf 0.34 (ethyl acetate). ^1^H-NMR-spectrum (DMSO-D_6_), δ, ppm: 2.85 (4H, s, 2 × CH_2_); 3.66 (4H, s, 2 × CH_2_); 4.79 (2H, s, CH_2_); 6.79 (2H, d, *J *= 7.9 Hz, H-2’, H-6’); 7.11 (2H, d, *J *= 7.4 Hz, H-3’, H-5’); 7.36 (2H, d, *J *= 8.2 Hz, H-3”, H-5”); 7.60 (2H, d, *J *= 7.9 Hz, H-2”, H-6”); 7.69 (1H, s, H-6); 11.47 (1H, s, N3H). ^13^C-NMR-spectrum (DMSO-D_6_), δ, ppm: 50.2; 50.6; 67.0; 118.8; 121.1; 122.5; 123.2; 124.5; 134.0; 134.1; 138.5; 149.8; 154.0; 154.2; 158.8; 160.1; 164.2.

**The general method for the synthesis of 1-[ω-(phenoxy)alkyl]-5-(morpholino)uracil (compounds 6–8)**.

A mixture of 4.61 mmol of 5-bromo-1-[ω-(phenoxy) alkyl]uracil and 1 ml (11.56 mmol) of morpholine was boiled in a solution of 50 ml of anhydrous ethylene glycol for 2 hours, cooled down to room temperature, 250 ml of cold water was added to the mixture, and it was left overnight at a temperature of 4 °C. The precipitate formed was filtered and recrystallized from ethyl acetate-hexane (3:1).

*1-[3-(Phenoxy)propyl]-5-(morpholino)uracil (6). *Yield 66%, *T*_mp_ 169–170°C, Rf 0.31 (ethyl acetate). ^1^H-NMR-spectum (DMSO-D_6_), δ, ppm: 2.04 (2H, q, *J *= 6.3 Hz, CH_2_); 3.77 (2H, t, *J *= 6.0 Hz, NCH_2_); 3.87 (2H, t, *J *= 5.7 Hz, CH_2_); 2.82 (4H, c, 2 × CH_2_); 3.69 (4H, c, 2 × CH_2_); 6.82–6.86 (3H, m, H-2’, H-4’, H-6’); 7.19 (2H, t, *J *= 8.0 Hz, H-3’, H-5’); 7.63 (1H, s, H-6); 11.37 (1H, s, N^3^H). ^13^C-NMR-spectrum (DMSO-D_6_), δ, ppm: 28.0; 45.4; 50.1; 50.5; 64.6; 100.9; 112.2; 122.3; 138.6; 145.8; 151.0; 158.4; 163.9.

*1-[4-(Phenoxy)butyl]-5-(morpholino)uracil (7). *Yield 78%, *T*_mp_ 156–159°C, Rf 0.65 (ethyl acetate). ^1^H-NMR-spectrum (DMSO-D_6_), δ, ppm: 1.64 (4H, s, CH_2_); 3.67 (2H, t, *J *= 6.2 Hz, CH_2_); 3.89 (2H, t, *J *= 6.2 Hz, CH_2_); 2.85 (4H, s, 2 × CH_2_); 3.66 (4H, s, 2 × CH_2_); 6.79–6.83 (3H, m, H-2’, H-4’, H-6’); 7.13 (2H, t, *J *= 8.1 Hz, H-3’, H-5’); 7.64 (1H, s, H-6); 11.41 (^1^H, s, N^3^H). ^13^C-NMR-spectrum (DMSO-D_6_), δ, ppm: 25.3; 25.7; 47.3; 50.4; 51.0; 66.8; 100.9; 114.4; 120.5; 129.5; 145.7; 151.0; 158.6; 165.8.

*1-[5-(Phenoxy)pentyl]-5-(morpholino)uracil (8). *Yield 71%, *T*_mp_ 162–163.5°C, Rf 0.78 (ethyl acetate). ^1^H-NMR-spectrum (DMSO-D_6_), δ, ppm: 1.39 (2H, q, *J *= 5.3 Hz, CH_2_); 1.63 (2H, q, *J *= 7.2 Hz, CH_2_); 1.72 (2H, q, *J *= 7.2 Hz, CH_2_); 3.67 (2H, t, *J *= 7.2 Hz, CH_2_); 3.93 (2H, t, *J *= 6.5 Hz, CH_2_); 2.88 (4H, s, 2 × CH_2_); 3.67 (4H, s, 2 × CH_2_); 6.83–6.88 (3H, m, H-2’, H-4’, H-6’); 7.22 (2H, t, *J *= 8.0 Hz, H-3’, H-5’); 7.55 (^1^H, s, H-6); 11.29 (^1^H, s, N^3^H). ^13^C-NMR-spectrum (DMSO-D_6_), δ, ppm: 22.9; 28.6; 28.7; 47.8; 50.5; 51.0; 67.5; 101.2; 114.8; 120.8; 129.9; 146.2; 151.4; 159.0; 164.3.

**Viruses**

A recombinant human type 5 adenovirus, expressing an enhanced green fluorescent protein (HAdV5-eGFP), was used in the study [19, 20].

**Cell culture**

HEK293 (human embryonic kidney) cells were used in the study [21]. HEK293 cells were cultivated in DMEM (Life Technologies UK) containing 10% fetal bovine serum (Life Technologies, UK), 4 mM *L*-glutamine, 1 mM sodium pyruvate, streptomycin/penicillin at a concentration of 100 μg/ml and 100 units/ml, respectively, at a temperature of 37 °C in an atmosphere of 5% CO_2_.

**MTT Assay**

HEK293 cells were incubated in the absence (control) or presence of various concentrations of the test compounds. After 24–72 h, 3-[4,5-dimethylthiazolyl-2-el]-2,5-diphenyltetrazolium bromide (MTT) was added to the cells; the final concentration of MTT was 0.5 mg/ml. After 2 hours of incubation at 37 °C, the living cells reduced the yellow MTT to dark-purple formazan granules. The culture medium was removed. The formazan granules were dissolved in DMSO, the amount of the reduced product was measured photometrically on a multi-function plate reader Synergy 2 Multi-Mode Reader (BioTek Instruments, USA) at 540 and 630 nm wavelengths [22].

**Resazurin Assay**

HEK293 cells were incubated in the absence (control) or presence of various concentrations of the test compounds. After 24–72 h, a resazurin dye (Sigma, USA) was added to the cells and was reduced by mitochondrial dehydrogenases of living cells to a fluorescent resarufin product (at excitation and emission wavelengths of 530 and 590 nm). Fluorescence intensity was recorded on a multi-function plate reader Synergy 2 Multi-Mode Reader (BioTek Instruments, USA).

**Determination of human adenovirus genome copy number**

To estimate the replication of HAdV5-eGFP 24 h after infection, the cells were harvested by trypsinization and total DNA was extracted with the QIAamp DNA Mini Kit (QIAGEN, Germany). Real-time qPCR was performed according to [23] on the CFX96™ Real-Time PCR Detection System (Bio-Rad, USA) using the iTaq™ Universal Probes Supermix reagent (Bio-Rad, USA).

**Titration of progeny viruses**

HEK293 cells were transduced with HAdV5-eGFP at multiplicities of infection of 1 and 10 PFU/cell. Three hours post transduction, solutions of the compounds **1 **and **3 **in DMSO at a concentration of 25 μM were added. DMSO was used as the control, and its final concentration in the culture medium did not exceed 0.1%. After 48 hours, the culture medium was collected in microtubes and frozen at -70 °C. To destroy cells, the virus-containing medium was thawed at room temperature and again frozen at -70 °C. After repeated thawing, aliquots of 10-fold dilutions of virus stocks were added to the HEK293 cells.

**Statistical analysis**

All data are presented as a mean ± standard deviation (SD). The statistical significance was determined using the GraphPad Prism 6 software (GraphPad Software, USA). A value of *p < *0.05 was considered statistically significant.

## RESULTS AND DISCUSSION

**Synthesis of the compounds**

In terms of chemical structure, the synthesized compounds are most similar to 1-benzyl-5-(arylamino)uracil derivatives [15]. These derivatives are active against human immunodeficiency virus type 1 (HIV-1) and the Epstein-Barr virus. We assumed that the 5-aminouracil and 5-amino-6-azauracil derivatives containing a substituent on N1 and analogous to the described compounds can exhibit inhibitory activity against DNA-containing viruses: in particular, adenoviruses.

**Fig. 1:**
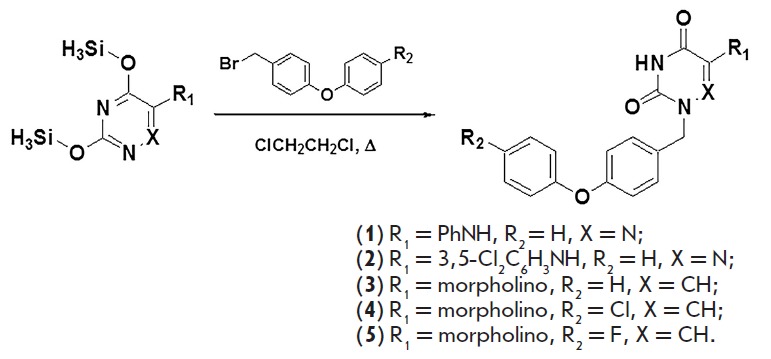
Scheme for the synthesis of 1-[4-(phenoxy)benzyl]- 5-(phenylamino)-6-azauracil (**1**), 1-[4-(phenoxy)benzyl]-5-[(3,5- dichlorophenyl)amino]-6-azauracil (**2**), and 1-[4-(phenoxy)benzyl]- (**3**), 1-[4-(4-chlorophenoxy) benzyl]-(**4**), 1-[4-(4-fluorophenoxy) benzyl]-5-(morpholino)uracil derivatives (**5**).

The synthesis of 1-[4-(phenoxy)benzyl]- 5-(phenylamino)-6-azauracil (**1**) and 1-[4-(phenoxy) benzyl]-5-[(3,5-dichlorophenyl)amino]-6-azauracil (**2**), as well as 1-[4-(phenoxy)benzyl]- (**3**), 1-[4-(4-chlorophenoxy)benzyl]- (**4**) and 1-[4-(4-fluorophenoxy) benzyl]-5-(morpholino)uracil (**5**) derivatives, was accomplished by condensation of 6-amino-3,5- bis(trimethylsilyloxy)-1,2,4-triazine or 5-amino-2,4- bis(trimethylsilyloxy)pyrimidine with an equimolar amount of the corresponding 4-(phenoxy)benzyl bromides through boiling in an anhydrous 1,2-dichloroethane solution in accordance with the previously described method [24]. The yield of compounds **1–5 **was 56-81% (*Fig. 1*).

**Fig. 2:**
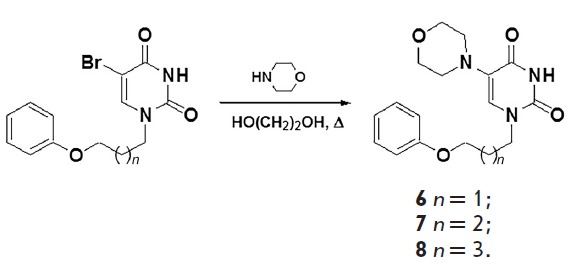
Scheme for the synthesis of 5-(morpholino)uracil derivatives **6–8
**

In order to study the relationships between the structure and antiviral activity, we synthesized analogues of the 5-(morpholino) derivative **3 **in which the 4-(phenoxy)benzyl fragment at N1 was replaced with a ω-(phenoxy)alkyl substituent. The synthesis of this group of compounds was carried out by amination of 5-bromo-1-[ω-(phenoxy)alkyl]uracil by morpholine through boiling in an ethylene glycol solution in accordance with the previously described method [15]. The yield of the target 5-(morpholino)uracil derivatives **6–8 **was 66–78% (*Fig. 2*).

**Cytotoxicity of the test compounds**

The cytotoxicity of the compounds was assessed by intravital staining of the HEK293 cells with MTT or trypan blue [25]. The test compounds in DMSO were added to the cells in a concentration range of 2.5–200 μM. The cells to which the appropriate amount of DMSO was added were used as a control.

**Table 1:** Anti-adenoviral activity of 5-aminouracil derivatives

Compound	IC_50_, μM^a^	TC_50_, μM^b^	SI^c^
1	9.2	53.6	5.8
2	0.5	47.6	95
3	8.7	103.1	11.9
4	13.1	64.8	4.9

^a^Concentration of half maximal inhibition at which the relative HAdV5-eGFP genome copy number is reduced by 50% compared to the control.

^b^bConcentration at which the number of living cells is reduced by 50%.

^c^cRatio of the compound TC_50_ to its IC_50_.

Intravital staining of the HEK293 cells by MTT was carried out 48 hours after the administration of the compounds. The toxicity of different doses of the compounds was determined by the viability of the cells compared to the control. All compounds at concentrations of up to 25 μM had no toxic effect on HEK293 cells. In addition, a concentration at which the number of living cells reduced by 50% was determined for the compounds showing inhibitory activity against human adenoviruses (TC50). To this end, the cells selectively stained with trypan blue were counted 48 hours after the addition of the compounds. The results are shown in *Table 1*.

**Fig. 3:**
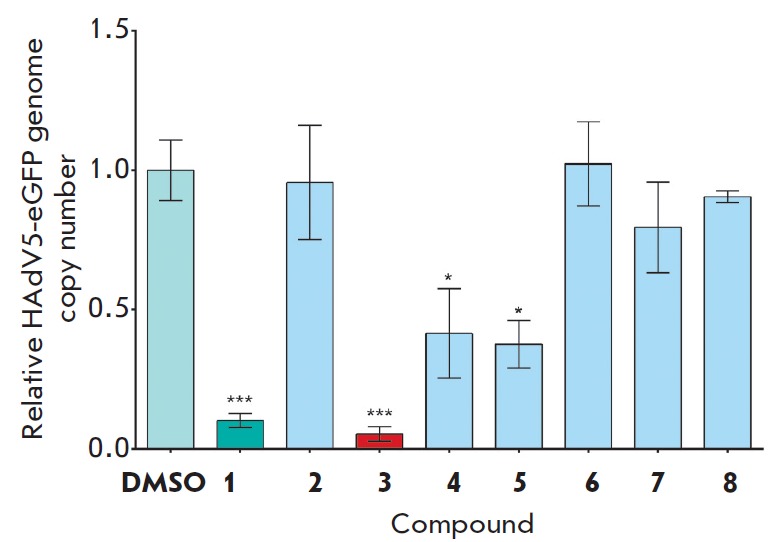
Assessment of the relative HAdV5-eGFP genome copy number in HEK293 cells treated with the test compounds. The differences between experimental and control samples were statistically significant at * *p < *0.05; *** *p* < 0.001.

**Anti-adenoviral activity of 5-aminouracil derivatives**

**Fig. 4:**
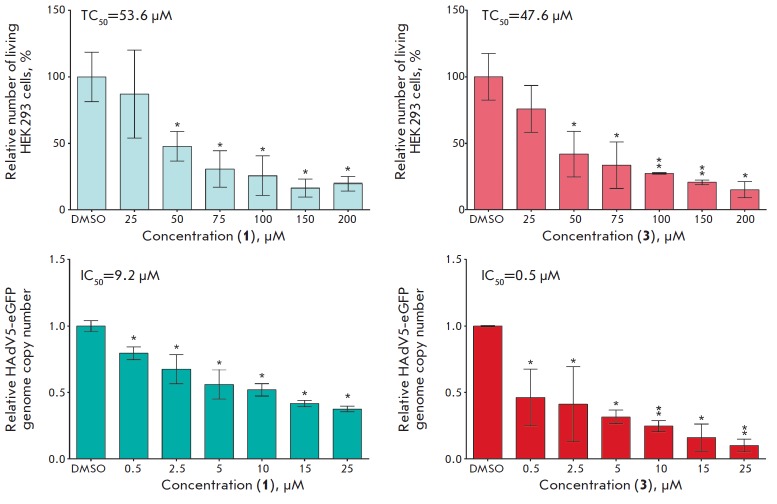
Anti-adenoviral activity of 5-aminouracil derivatives. TC50 and IC_50_ of compounds **1 **(*A*) and **3 **(*B*). Differences between experimental and control samples were statistically significant at * *p* < 0.05; *** *p* < 0.001.

**Anti-adenoviral activity of 5-aminouracil derivatives**

During the evaluation of the anti-adenoviral activity of 5-aminouracil derivatives, the HEK293 cells were transduced with recombinant type 5 human adenovirus expressing the enhanced green fluorescent protein HAdV5-eGFP with a multiplicity of infections of 1 PFU/cell. The test compounds were added to the cells at a concentration of 25 μM 3 h post-transduction to give the recombinant adenovirus enough time to undergo the initial stage of the replication cycle (the interaction of the virus with cell surface receptors and penetration into the cell). DMSO was used as a negative control. The concentration of DMSO in all samples did not exceed 0.1%. To assess the inhibitory activity of the compounds, newly synthesized HAdV5-eGFP genomes were detected via real-time qPCR 24 hours later [23]. It has been shown that compounds **1**, **3**, **4 **and **5 **display marked inhibitory activity with respect to HAdV5-eGFP replication (*Fig. 3*).

**Fig. 5:**
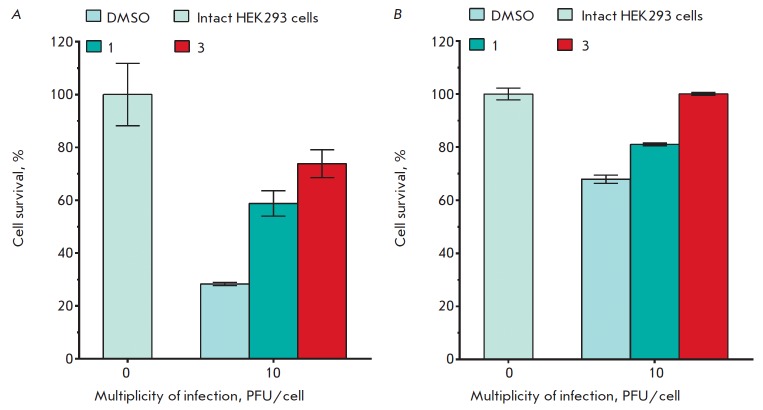
The survival of HEK293 cells transduced with HAdV5-eGFP and treated with the 5-aminouracil derivatives **1 **and **3**. *(A) *The results were obtained using the MTT assay. 100% corresponds to optical density value of intact HEK293 cells (control sample). All reported values are means of three independent measurements with standard deviations. The differences between experimental and control samples were statistically significant in all cases (*p * < 0.05). (*B*) Data was obtained using the resazurin assay. One hundred percent corresponds to the fluorescence intensity of intact HEK293 cells (control sample). All reported values are means of three independent measurements with standard deviations. The differences between DMSO samples and other samples were statistically significant in all cases (*p* < 0.05). The differences between intact cells samples and samples of group “3” were statistically insignificant.

A concentration corresponding to the half-maximal inhibition (IC_50_) at which the relative HAdV5-eGFP genome copy number was reduced by 50% compared to the control was determined for compounds **1**, **3**, **4 **and **5**, which display inhibitory activity against human adenovirus. HEK293 cells were transduced by HAdV5- eGFP with a multiplicity of infections of 1 PFU/cell. Three hours post-transduction, the test compounds were added to the cells at concentrations of 0.5, 2.5, 5, 10, 15 and 25 μM. The concentration of DMSO in all samples did not exceed 0.1%. The inhibitory activity of the compounds was assessed 24 hours later by the determination of the HAdV5-eGFP genome copy number via qPCR (*Fig. 4*). The selectivity index (SI) was calculated as the ratio of TC50 of the compound to its IC_50_ (*Table 1*). These quantitative indices of inhibition can be used as a measure of the tested compounds’ effectiveness; i.e., the degree of suppression of HAdV5-eGFP replication in HEK293 cells.

**Table 2:** Structures of K_V_-channels alone and in complex with charybdotoxin used in homology modeling studies

Multiplicity of infection	Compound		
	DMSO	1	2
MOI 1	1 × 10^4^	5.1 × 10^3^	2.3 × 10^3^
MOI 10	2.7 × 10^6^	1.7 × 10^5^	3.7 × 10^5^

It has been demonstrated that the most potent antiviral effect is exhibited by 5-(morpholino)-derivative **3 **with IC_50_ of 0.5 μM, and SI = 95. The 6-azauracil derivatives were either an order of magnitude less active (compound **1**) or did not display any inhibitory properties at all (compound **2**). It has also been shown that the introduction of a chlorine atom (compound **4**) or a fluorine atom (compound **5**) into the *para*-position of the 4-(phenoxy)benzyl moiety significantly reduces inhibitory activity. At the same time, the replacement of benzyl in the 4-(phenoxy)benzyl moiety by an aliphatic chain leads to compounds **6–8 **which have no anti-adenoviral activity. This fact indicates the high importance of the aromatic fragment in the antiviral properties of series of the tested compounds.

In addition, the impact of the most effective 5-aminouracil derivatives, compounds **1 **and **3, **on the production of progeny infectious HAdV5-eGFP was evaluated. A decrease in progeny virus titer was observed for these compounds (*Table 2*).

Based on the data presented, it can be assumed that the mechanism of action of the series of tested compounds is associated with the inhibition of viral replication key factors, i.e. viral DNA polymerase and products of the *E1A* gene [26, 27].

During the experiment, survival of the HEK293 cells infected with HAdV5 at a multiplicity of infection of 10 PFU/cell in the presence of compounds **1 **and **3 **was also assessed (*Fig. 5*). Three hours post infection, solutions of the compounds **1 **and **3 **in DMSO at a concentration of 25 μM were added to the cells. According to the MTT assay, cell survival 48 hours post infection at a multiplicity of 10 PFU/cell was 74 and 59% in the presence of compounds **1 **and **3**, respectively, compared to the control. These data are consistent with the results obtained in a similar analysis of cell survival during adenovirus infection (MOI 10 PFU/cell) using resazurin. For example, after exposure to compounds **1 **and **3**, the proportion of living cells did not differ significantly from the proportion in the control sample (*Fig. 5*). The obtained data indicate that the test compounds possess antiviral properties.

## CONCLUSION

Thus, a new type of anti-adenoviral agents of a nonnucleoside nature has been discovered that exhibit an inhibitory effect on human adenoviruses. Compounds of this series may be promising candidates for the development of drugs effective against adenoviral infections.

## Abbreviations

HAdV: human adenovirus
HIV: human immunodeficiency virus
HMDS: hexamethyldisilazane
